# Long-Term Survival Achieved with Multimodal Therapy and Multiple Hepatic Resections for Pancreatic Primary Mixed Neuroendocrine–Non-Neuroendocrine Neoplasm Comprising Acinar Cell Carcinoma and Neuroendocrine Carcinoma: A Case Report

**DOI:** 10.70352/scrj.cr.25-0279

**Published:** 2025-11-07

**Authors:** Hiroto Arino, Tatsuki Ishikawa, Tomoe Kimura, Tatsuya Koyama, Kai Nakao, Yusuke Okamura, Masashi Saji, Nobu Oshima, Masato Narita, Masato Kondo, Kenji Uryuhara, Hiroyuki Kobayashi, Shigeo Hara, Satoshi Kaihara

**Affiliations:** 1 Department of Surgery, Kobe City Medical Center General Hospital, Kobe, Hyogo, Japan; 2 Department of Pathology, Kobe City Medical Center General Hospital, Kobe, Hyogo, Japan

**Keywords:** mixed neuroendocrine–non-neuroendocrine neoplasm (MINEN), acinar cell carcinoma (ACC), neuroendocrine carcinoma (NEC)

## Abstract

**INTRODUCTION:**

In the 2019 World Health Organization (WHO) classification, tumors comprising ≥30% of both neuroendocrine and non-neuroendocrine components are defined as mixed neuroendocrine–non-neuroendocrine neoplasms (MiNENs). Acinar cell carcinoma (ACC) constitutes <1% of pancreatic tumors, while neuroendocrine carcinoma (NEC) accounts for 1%–2%, making MiNENs with both characteristics extremely rare. The role of surgical resection in resectable NEC remains unclear; however, it is generally recommended as part of multimodal therapy. This case presents a pancreatic mixed acinar–NEC successfully managed with multimodal therapy, including repeated liver metastasis resections, achieving long-term disease control.

**CASE PRESENTATION:**

A 57-year-old woman presented with abdominal pain. Her symptoms improved with conservative treatment, but the patient returned to the emergency room 3 months later with abdominal pain. Hemorrhage from a pancreatic tail cyst and localized pancreatitis were suspected based on laboratory and imaging findings. As malignancy could not be ruled out, the patient was referred to our department and underwent laparoscopic distal pancreatectomy with splenectomy. Pathological examination revealed mixed acinar–NEC composed of 70% ACC and 30% NEC; later, liver metastasis was detected. While progression was observed after cisplatin plus etoposide combination therapy, stereotactic body radiation therapy led to a gradual reduction in the size of the liver tumor. Six months postoperatively, laparoscopic partial hepatectomy was performed. Despite adjuvant chemotherapy, a solitary liver tumor from ACC was detected 1 month after surgery. After FOLFIRINOX chemotherapy administration, another laparoscopic partial hepatectomy was performed. No recurrence has since been observed.

**CONCLUSIONS:**

In cases where disease control is achieved via multimodal therapy, surgical resection of metastases may be considered to provide long-term survival.

## Abbreviations

ACC
: acinar cell carcinoma
MiNEN
: mixed neuroendocrine–non-neuroendocrine neoplasms
NCCN
: National Comprehensive Cancer Network
NEC
: neuroendocrine carcinoma
NEN
: neuroendocrine neoplasms
SBRT
: stereotactic body radiation therapy

## INTRODUCTION

In the 2019 World Health Organization (WHO) classification, MiNENs are defined as tumors comprising ≥30% of both neuroendocrine and non-neuroendocrine components.^1)^ The incidence of ACC in pancreatic tumors is <1%.^2,3)^ NEC accounts for 1%–2%.^4)^ MiNENs possessing both ACC and NEC characteristics are extremely rare. The role of surgical resection for resectable NEC remains unclear; however, it is considered part of a multimodal treatment approach.^5–7)^

## CASE PRESENTATION

**Fig. 1:**
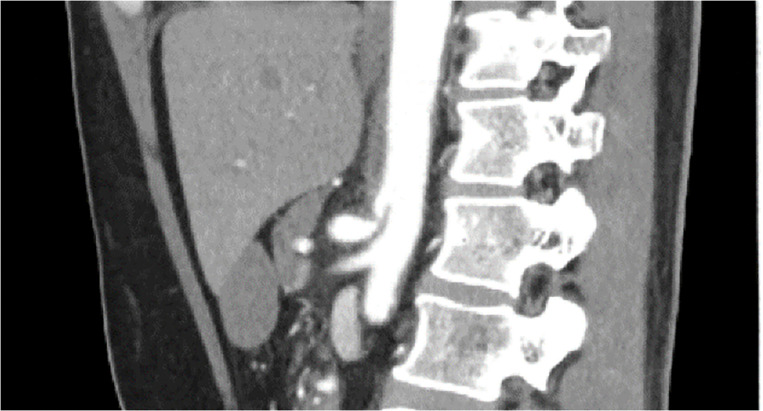
Contrast-enhanced CT on initial admission showing the primary pancreatic lesion.

A 57-year-old woman with a history of cesarean section and dyslipidemia presented with abdominal pain in month Y, year X. She was admitted with suspected median arcuate ligament syndrome (**Fig. 1**). Her symptoms improved with conservative treatment, including pain and blood pressure control, and she was discharged on the 9th day of hospitalization.

**Fig. 2:**
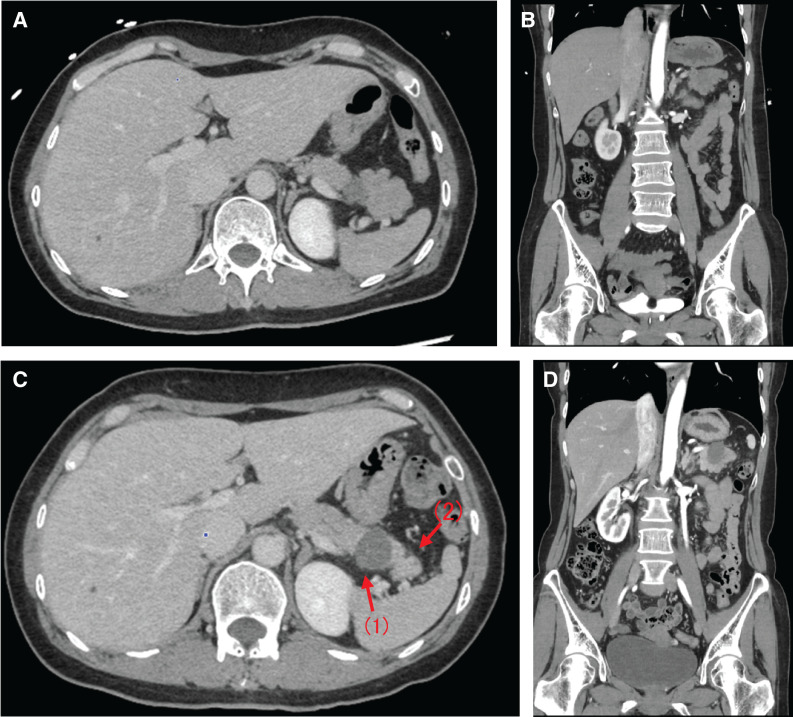
(**A**, **B**) Contrast-enhanced CT images of the pancreatic lesion at initial admission. (**C**, **D**) CT images of the pancreatic lesion on 2nd admission, revealing progression in size and morphology (arrows).

In month Y + 3, year X, the patient returned to the emergency department with abdominal pain. Hemorrhage from a pancreatic tail cyst and localized pancreatitis were suspected based on laboratory and imaging findings, leading to hospitalization. CT showed rapid enlargement of a pancreatic tail mass that had been previously noted in month Y (**Fig. 2**).

(1) The CT value of the cyst was slightly elevated, suggesting a cyst containing hemorrhage or high-protein components, and (2) the distal pancreatic parenchyma was possibly affected by pancreatitis due to the increased density of the surrounding adipose tissue.

Although repeated pancreatic juice cytology did not reveal malignancy, malignancy could not be ruled out. Therefore, the patient was referred to our department for diagnostic treatment in month Y + 5 and underwent laparoscopic distal pancreatectomy with splenectomy in month Y + 6.

**Fig. 3:**
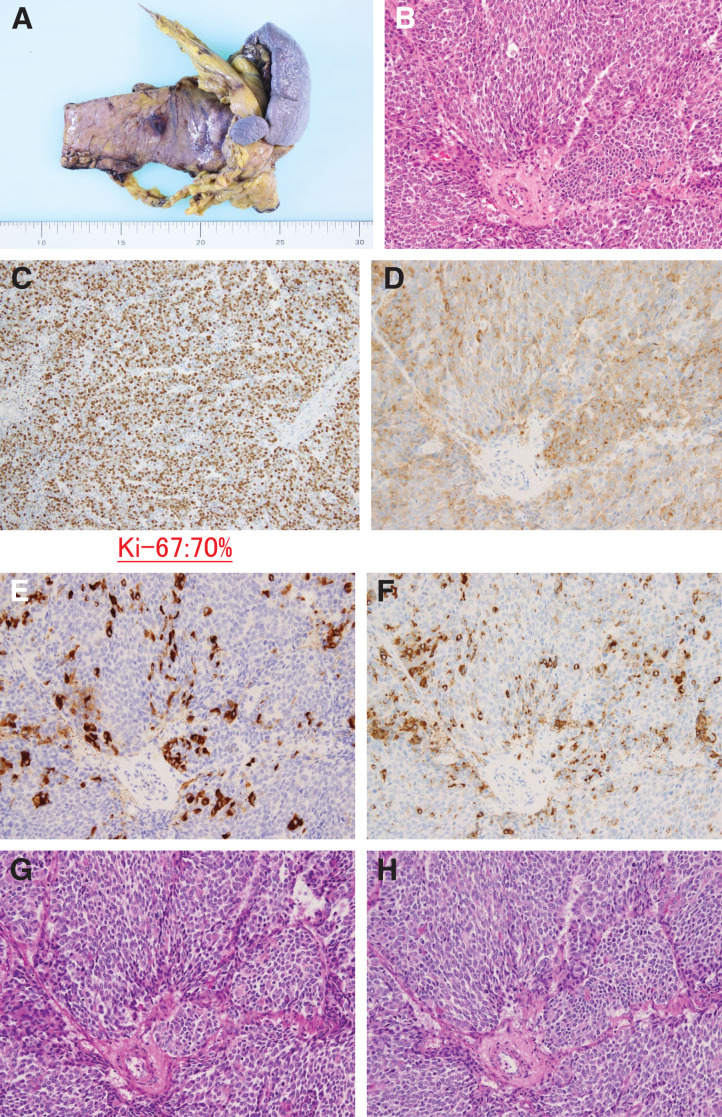
(**A**) Surgical specimen obtained during the initial operation, including the pancreatic body, tail, and spleen. (**B**) HE staining of the pancreatic tumor (×200). (**C**) Ki-67 immunostaining (×100) showing a high proliferation index. (**D**) Synaptophysin staining (×200), (**E**) Bcl-10 staining (×200), and (**F**) chromogranin A staining (×200). (**G**) dPAS staining and (**H**) PAS staining. dPAS, diastase-Periodic acid–Schiff; HE, Hematoxylin and eosin; PAS, Periodic acid-Schiff

**Fig. 4:**
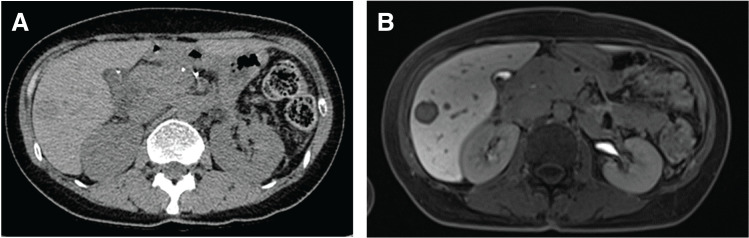
(**A**) CT image of the S6 segment metastatic liver tumor identified at 7 months after the initial surgery. (**B**) Gd-EOB-DTPA-enhanced MRI (late phase) showing the same lesion. Gd-EOB-DTPA, gadolinium ethoxybenzyl diethylenetriamine pentaacetic acid-enhanced

**Fig. 5:**
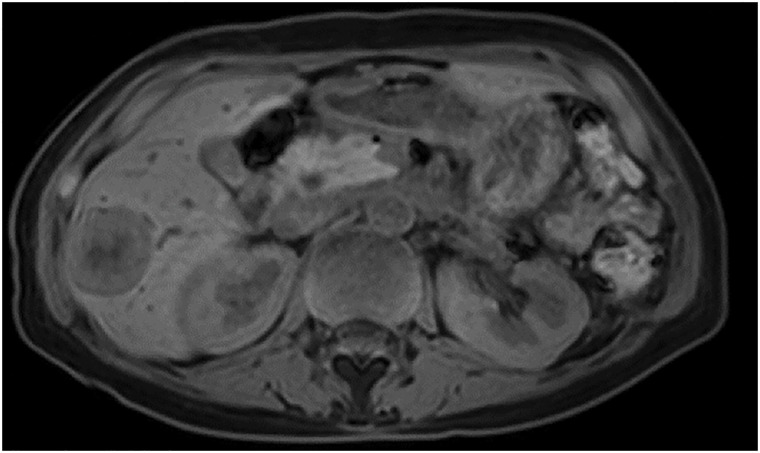
Late-phase Gd-EOB-DTPA-enhanced MRI of the S6 metastatic liver tumor after 3 courses of chemotherapy, showing a partial response. Gd-EOB-DTPA, gadolinium ethoxybenzyl diethylenetriamine pentaacetic acid

**Fig. 6:**
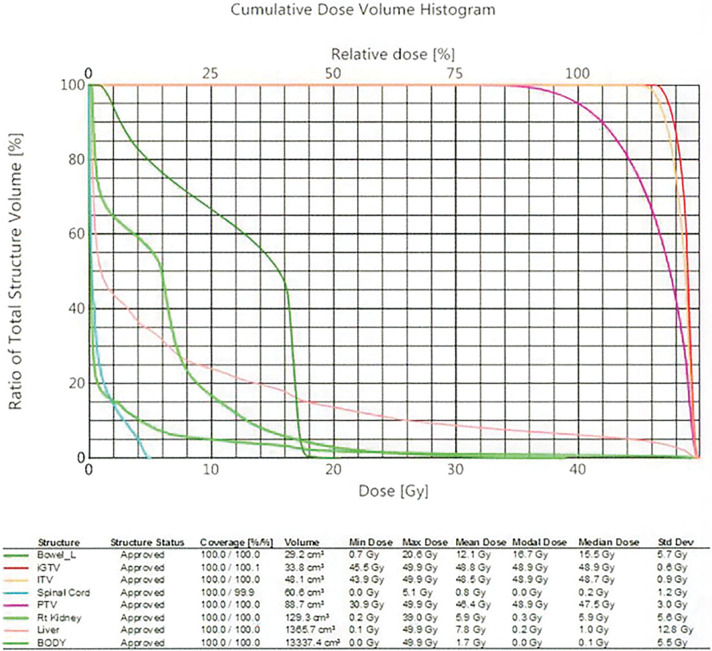
SBRT dosimetric details, including total dose, fractionation, PTV margins, and OAR constraints. iGTV, internal gross tumour volume; ITV, internal target volume; OAR, organs at risk; PTV, planning target volume; Rt, right; SBRT, stereotactic body radiation therapy

**Fig. 7:**
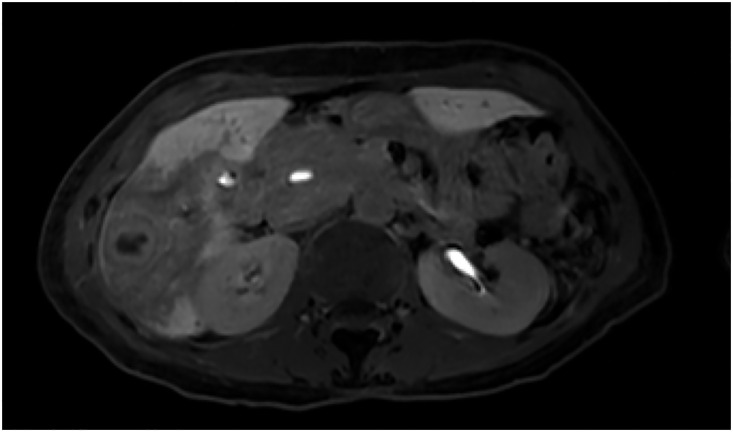
Late-phase Gd-EOB-DTPA-enhanced MRI of the S6 metastatic liver tumor after SBRT, showing further tumor regression. Gd-EOB-DTPA, gadolinium ethoxybenzyl diethylenetriamine pentaacetic acid; SBRT, stereotactic body radiation therapy

**Fig. 8:**
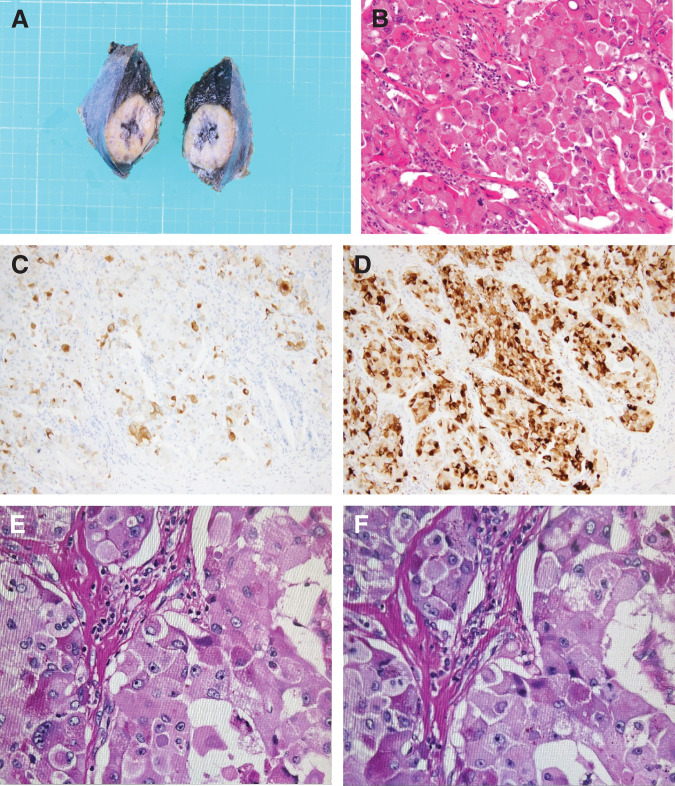
(**A**) Resected specimen from the S6 metastatic liver tumor. (**B**) HE staining of the tumor (×200). (**C**) Synaptophysin staining (×100). (**D**) Bcl-10 staining (×100), confirming neuroendocrine differentiation. (**E**) dPAS staining and (**F**) PAS staining. dPAS, diastase-Periodic acid–Schiff; HE, hematoxylin and eosin; PAS, periodic acid-Schiff

Pathological examination revealed mixed acinar–NEC composed of 70% ACC and 30% NEC (pT3N1aM0; pStage IIB) (**Fig. 3**). In month Y + 7, a solitary liver metastasis (S6) was detected (**Fig. 4**), and liver biopsy confirmed it as a metastasis of mixed acinar–NEC. The biopsy specimen was diagnosed as metastatic NEC. Cisplatin plus etoposide combination therapy was initiated; however, progression was observed after 3 courses (**Fig. 5**). Stereotactic body radiation therapy was performed in month Y+10 (40 Gy/5 fractions), leading to a gradual reduction in the size of the liver tumor (**Fig. 6**). Nine months postoperatively, with only the solitary liver metastasis under control (**Fig. 7**), laparoscopic partial hepatectomy (S6) was performed in month Y + 15. Pathological examination revealed that the liver metastasis was predominantly composed of ACC (**Fig. 8**).

**Fig. 9:**
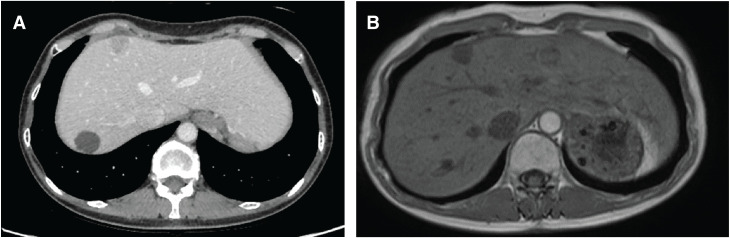
(**A**) CT image showing a new metastatic liver tumor in segment S4 at 10 months post-initial surgery. (**B**) Gd-EOB-DTPA-enhanced MRI (late phase) demonstrating the tumor’s hypervascular nature. Gd-EOB-DTPA, gadolinium ethoxybenzyl diethylenetriamine pentaacetic acid

**Fig. 10:**
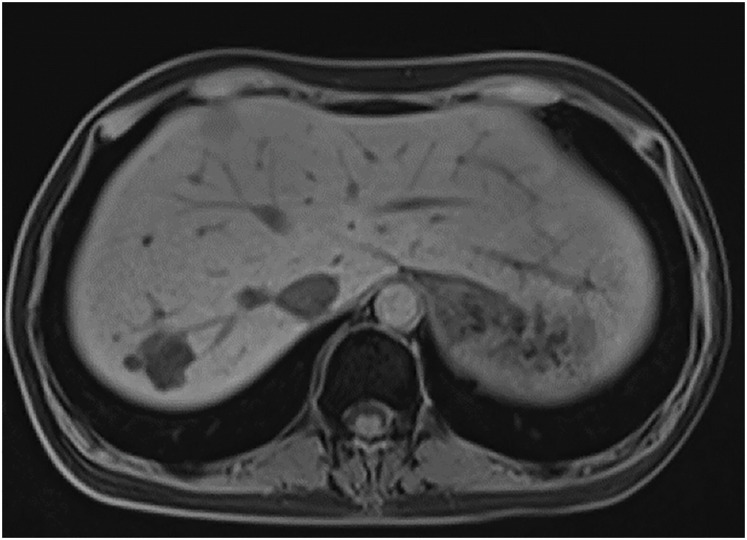
Late-phase Gd-EOB-DTPA-enhanced MRI of the S4 metastatic liver tumor after 10 courses of modified FOLFIRINOX chemotherapy, indicating a favorable therapeutic response. Gd-EOB-DTPA, gadolinium ethoxybenzyl diethylenetriamine pentaacetic acid

**Fig. 11:**
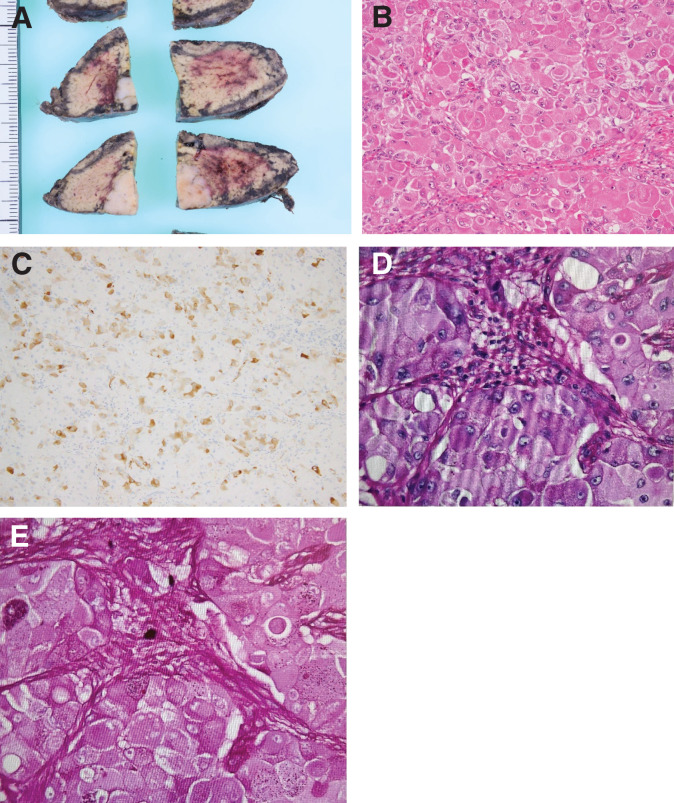
(**A**) Surgical specimen from the S4 metastatic liver resection. (**B**) HE staining of the tumor (×200). (**C**) Synaptophysin staining (×100), confirming persistence of neuroendocrine features. (**D**) dPAS staining and (**E**) PAS staining. dPAS, diastase-Periodic acid–Schiff; HE, hematoxylin and eosin; PAS, periodic acid-Schiff

**Fig. 12:**
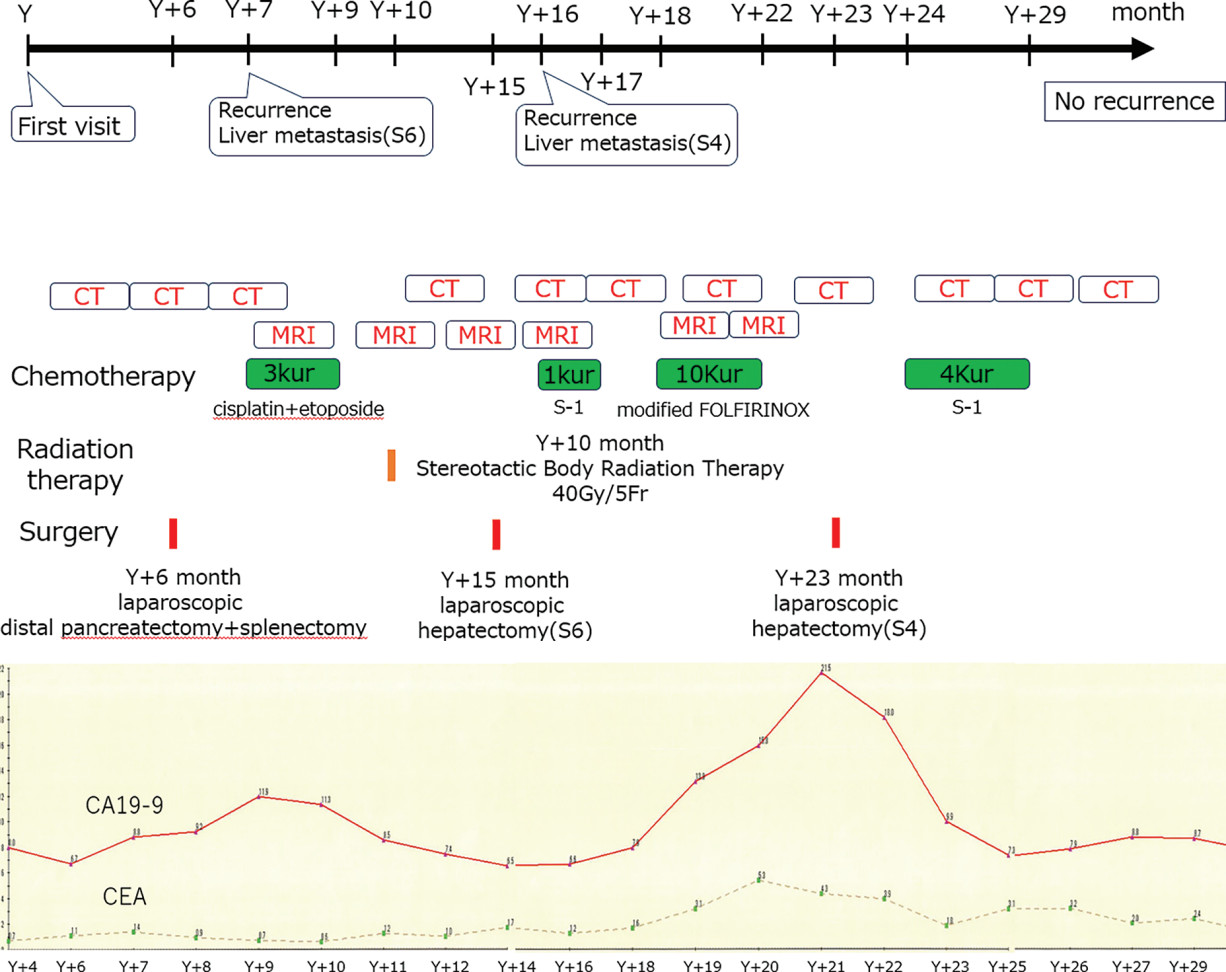
Treatment course including surgery, chemotherapy, surveillance imaging frequency, tumor markers, and radiotherapy. CA19-9, carbohydrate antigen 19-9; CEA, carcinoembryonic antigen

Adjuvant chemotherapy with S-1 was initiated in month Y + 16; however, a solitary liver tumor in S4 was detected 1 month after surgery. Liver biopsy diagnosed the lesion as a metastatic liver tumor from ACC (**Fig. 9**). Modified FOLFIRINOX chemotherapy was administered for 10 courses as 2nd-line treatment. As the disease was stable upon evaluation (**Fig. 10**), laparoscopic partial hepatectomy (S4) was performed in month Y + 23. Pathological findings confirmed the metastatic liver tumor as predominantly ACC in mixed acinar–NEC (**Fig. 11**). Postoperative S-1 was administered for 6 months. As of month Y + 37, the patient remains disease-free (**Fig. 12**).

## DISCUSSION

Owing to the rarity of mixed acinar–NEC, no established treatment guidelines exist, and it is generally managed according to NEC protocols. Gastroenteropancreatic NEC (GEP-NEC) and small cell lung cancer share morphological similarities, genetic abnormality profiles, and clinical courses. Given the difficulty of conducting clinical trials for infrequent GEP-NEC, evidence from small cell lung cancer is often used in its management. Curative surgical resection is less significant in GEP-NEC compared with cancers of the same organ. However, GEP-NEC is highly sensitive to chemotherapy and radiation, suggesting that these treatments should be prioritized over surgical resection. The Japanese NEN clinical practice guidelines outline distinct treatment recommendations for esophageal NEC, non-esophageal gastrointestinal NEC, and pancreatic NEC. However, the indications and significance of surgery, even for resectable cases, are not clearly defined.^5)^

The European Society for Medical Oncology guidelines explicitly state that upfront surgery for locally advanced or metastatic NEC is not recommended, whereas the NCCN guidelines suggest surgical resection as an option for resectable NEC, depending on the primary organ’s location, in combination with pre- or postoperative chemotherapy (± radiation therapy). However, surgical resection is not mentioned for unresectable locoregional NEC.^6,7)^ Chemotherapy for GEP-NEC has a high response rate, exceeding 50%. Surgical resection after tumor shrinkage via neoadjuvant chemotherapy aligns with clinical practice and is considered an acceptable treatment strategy.^8–11)^

In this case, tumor resection was performed for diagnostic treatment, followed by combined postoperative chemotherapy and radiation therapy. The chemotherapy regimen was selected based on the histological type. Surgical resection was subsequently performed as part of a multidisciplinary approach. Cisplatin plus etoposide, a chemotherapy regimen used for small cell lung cancer, was selected for treatment. Subsequently, a prospective trial’s subgroup analysis comparing the efficacy of cisplatin plus etoposide versus cisplatin plus irinotecan reported better overall survival outcomes for pancreatic NEC with the cisplatin plus etoposide regimen.^12)^

Radiation therapy is used as adjuvant treatment for resectable cases or concurrently/sequentially with chemotherapy for locally advanced NEC, according to NCCN guidelines.^6)^ Mixed acinar–NEC composed of ACC and NEC has a poorer prognosis than ACC alone.^13,14)^ Disseminated recurrence is common even after complete resection. Although some cases of mixed acinar–NEC have survived after surgical resection of solitary liver metastases followed by chemotherapy and primary tumor resection,^15)^ no reports have documented disease-free survival achieved via multiple surgeries for metastases.

## CONCLUSIONS

Most cases possess either unresectable metastases or become refractory to multimodal treatment excluding surgery. In cases where disease control is achieved via multimodal therapy, as in the present case, surgical resection of metastases may be considered. Surgical resection of metastatic mixed acinar–NEC controlled by multimodal therapy may provide long-term survival.

## Data Availability

The data generated or analyzed during this study are included in this published article.
